# 

**Published:** 1891-03

**Authors:** 


### CONTENTS.

ORIGINAL COMMUNICATIONS—

Adenomata of the Pharynx, with Report of Cases. By L. M. Crichton, M. D., Atlanta, Ga. .... 1

Circumscribed Traumatic Aneurism of a Tibial Artery. By Howard J. Williams, A. M., M. D. .... 9

A Plea for Higher Medical Education. By A. B. Patterson, M. D., Atlanta, Ga. .... 14

Mercury. By J. S. Todd, M. D. .... 16

One Hundred Consecutive Cases of Skin Disease, III. By M. B. Hutchins, M. D. .... 33

SOCIETY REPORTS—

Gynecological and Obstetrical Society of Baltimore .... 37

The Philadelphia Electro-Therapeutic Society .... 47

BOOK REVIEWS—

Science and Art of Obstetrics .... 51

An Encyclopedic Medical Dictionary .... 51

EDITORIAL—

Recent Graduates .... 52

Announcement .... 53

Our Sympathy .... 53

Items .... 50, 54–64

